# High-confidence assessment of functional impact of human mitochondrial non-synonymous genome variations by APOGEE

**DOI:** 10.1371/journal.pcbi.1005628

**Published:** 2017-06-22

**Authors:** Stefano Castellana, Caterina Fusilli, Gianluigi Mazzoccoli, Tommaso Biagini, Daniele Capocefalo, Massimo Carella, Angelo Luigi Vescovi, Tommaso Mazza

**Affiliations:** 1 IRCCS Casa Sollievo della Sofferenza, Bioinformatics unit, San Giovanni Rotondo (FG), Italy; 2 IRCCS Casa Sollievo della Sofferenza, Department of Medical Sciences, Division of Internal Medicine, San Giovanni Rotondo (FG), Italy; 3 IRCCS Casa Sollievo della Sofferenza, Medical Genetics unit, San Giovanni Rotondo (FG), Italy; 4 IRCSS Casa Sollievo della Sofferenza, ISBReMIT- Institute for Stem Cell Biology, Regenerative Medicine and Innovative Therapies, San Giovanni Rotondo (FG), Italy; 5 University of Milano Bicocca, Department of Biotechnology and Biosciences, Milan, Italy; Rutgers University, UNITED STATES

## Abstract

24,189 are all the possible non-synonymous amino acid changes potentially affecting the human mitochondrial DNA. Only a tiny subset was functionally evaluated with certainty so far, while the pathogenicity of the vast majority was only assessed *in-silico* by software predictors. Since these tools proved to be rather incongruent, we have designed and implemented APOGEE, a machine-learning algorithm that outperforms all existing prediction methods in estimating the harmfulness of mitochondrial non-synonymous genome variations. We provide a detailed description of the underlying algorithm, of the selected and manually curated training and test sets of variants, as well as of its classification ability.

## Author summary

The mitochondrion is an organelle floating in the cytoplasm of almost all eukaryotic cells. Its primary function is to generate energy. It contains an independent DNA (mtDNA), which is inherited maternally in many organisms. This DNA is highly susceptible to mutations since it does not possess the robust DNA repair mechanisms proper of the nuclear DNA. Mutations in the mtDNA were associated to several inherited and acquired mitochondrial diseases, including Alzheimer and Parkinson diseases, and cancer. The assessment of the mutation-disease causal link is an onerous task. It requires important laboratory skills/equipment and, often, an animal facility, which are not always available to any laboratory altogether. More and more often, one falls back on software solutions that rely on structural and functional characteristics of proteins to predict the putative harmfulness of a mutation. Many have been implemented and tested on the nuclear proteins, but only a few were finely tuned to the “neglected genome”. Our work not only presents APOGEE, a machine-learning-based predictor that outperforms all existing predictors in reliability and sensitivity, but it makes freely available the APOGEE’s predictions for all the mitochondrial missense mutations in MitImpact.

## Introduction

Assessing the pathogenicity of genome mutations is a notoriously onerous task both *in-vitro* and *in-vivo*, and occasionally even unviable because of the paucity of funds or of proper analytical facilities. This is particularly true when dealing with the mitochondrial DNA, which is less studied, although significantly smaller, than the nuclear counterpart [1]. This task was massively faced from a computational point of view though, and a growing number of algorithms and software packages, which elaborate sequence, structural and functional data to yield plausible evaluations of the harmfulness of variant amino acids in the form of pathogenicity scores and categorical, often dichotomous, variables, were implemented and released over time.

Their assessments of pathogenicity are actual predictions, whose global congruency was deeply investigated by a few comparative studies [2–7]. Generally, only 60–70% agreement resulted when considering all the possible human non-synonymous variants. No single predictor emerged, neither in terms of classification accuracy, nor of specificity and sensitivity [6]. Similar results were achieved when considering only a subset of 173 validated disease-causing mitochondrial mutations taken from MITOMAP [8]: 64% were deemed *possibly* or *probably damaging* by PolyPhen-2, 62% as being *high* or *medium impact* variants by MutationAssessor and 61% as being *deleterious* by PROVEAN. The worst performance was achieved by SIFT, with only 16% of true positives, and by FatHmm that correctly classified only one variant on 173. Even with this subset of validated mutations or with those falling in ultraconserved genomic loci, predictions were broadly incongruent. Reasons for that were ascribed to the intrinsic differences between computational/statistical methods and reference databases, or between training datasets and alignment algorithms [2–7]. These facts drove the development of the so called *aggregators* or *meta-predictors*, namely those software packages that yield an evaluation of pathogenicity based on the outcomes of other reference predictors, as well as of databases of nuclear and mitochondrial precomputed predictions [9–12]. Even these were contrasting [2].

Due to their high incongruence and since almost all existing predictors were tailored to the nuclear genome, which is an important contributing factor to their modest classification performance and incongruence, we designed APOGEE. It grounds on three milestones: it feeds third-party predictors with features that are strictly related to the 13 mitochondrial proteins, like multi-alignments and amino acids conservation estimates; its reasoning strategy was tuned on finely curated, non-overlapping, training sets of variations; its predicting model was based on *decision tree learning* in order to provide investigable rules of pathogenicity.

## Results

The classification engine of APOGEE was built on 100 sets of variants drawn randomly and with replacement from a training set of 864 known mitochondrial variants. This strategy left out-of-bag as many test sets of variants on which we calculated an array of performance metrics. These were additionally calculated for all aggregated individual predictors and were reported in Table 1. It is important to notice that our training set overlaps those used by most of the aggregated software predictors, which are available from http://structure.bmc.lu.se/VariBench/GrimmDatasets.php, of only 102 on 864 variants.

**Table 1:** Performance evaluation calculated on 864 known mitochondrial non-synonymous variants. Number of available predictions in last column.

**	*TP*	*TN*	*FP*	*FN*	*Specificity* *TN/(FP+TN)*	*Sensitivity* *TP/(TP+FN)*	*Accuracy* *(TP+TN)/(P+N)*	*Precision* *TP/(TP+FP)*	*FDR* *FP/(TP+FP)*	*MCC*	*MCR*	*N Predicted*
PolyPhen2^*^	120	369	263	100	0,58	0,54	0,57	0,31	0,68	0,11	42,61	852
*PolyPhen2b^#^*	139	288	344	81	0,46	0,63	0,51	0,29	0,71	0,08	49,88	852
*SIFT*	31	560	81	191	0,87	0,14	0,68	0,28	0,72	0,02	31,52	863
*FatHmm*	0	638	3	222	0,99	0,00	0,74	0,00	1,00	-0,03	26,07	863
*FatHmm_W*	82	473	168	141	0,74	0,39	0,64	0,33	0,67	0,11	35,76	863
*PROVEAN*	128	329	312	94	0,51	0,58	0,53	0,29	0,71	0,08	47,05	863
*MutationAssessor^§^*	130	331	307	87	0,52	0,59	0,54	0,29	0,70	0,11	46,08	854
*EFIN 1 (HD)*	69	432	79	154	0,84	0,31	0,68	0,47	0,53	0,18	31,74	734
*EFIN 2 (SP)*	83	511	130	140	0,79	0,37	0,69	0,39	0,61	0,17	31,25	864
*CADD*	69	495	146	154	0,77	0,31	0,65	0,32	0,68	0,08	34,72	864
*PANTHER*	89	353	213	102	0,62	0,47	0,58	0,29	0,71	0,08	41,61	757
*PhD-SNP*	141	291	350	82	0,45	0,63	0,51	0,28	0,71	0,07	50,00	864
*SNAP*	128	345	296	95	0,54	0,57	0,55	0,30	0,69	0,09	45,25	864
***Meta-predictors***												
*MetaSNP*	128	340	301	95	0,53	0,57	0,54	0,29	0,71	0,09	45,83	864
*CAROL*	117	399	242	106	0,62	0,52	0,59	0,33	0,67	0,13	40,28	864
*Condel*	85	337	304	138	0,53	0,38	0,49	0,23	0,78	-0,08	51,16	864
*COVEC WMV*	117	374	242	103	0,61	0,53	0,59	0,33	0,67	0,12	41,27	836
*MToolBox DS*	142	276	365	81	0,43	0,64	0,48	0,28	0,72	0,06	51,62	864
***APOGEE Bootstrap***	**162**	**564**	**61**	**77**	**0,9**	**0,68**	**0,84**	**0,73**	**0,27**	**0,59**	**15,97**	**864**

^*^possibly damaging variants considered as benign

^#^*possibly damaging* variants considered as harmful

^§^*low* and *neutral* predictions considered as harmless, while *medium* and *high impact* predictions are considered pathogenic.

Performance of the considered predictors were generally low, with elevate misclassification rates (MCRs) and low Matthew’s Correlation Coefficient (MCCs) for all the investigated methods, but EFIN that achieved good specificity, accuracy and relatively low MCR. FatHmm_W outperformed FatHmm, both in terms of sensitivity and precision. CADD and FatHmm MCRs were also sensibly low. Among the meta-predictors, CAROL and COVEC WMV, which assembled only two (SIFT and PolyPhen2) and three (SIFT, PolyPhen2 and MutationAssessor) primary scores, respectively, showed decent performances. Pairwise comparisons of predictions revealed good agreement between all individual tools, but PANTHER that was mostly discordant (S1 Text). On the contrary, the outcomes of the meta-predictors were generally poorly congruent. In particular, Condel was mostly in disagreement with all the others (S1 Text).

**Fig 1:**
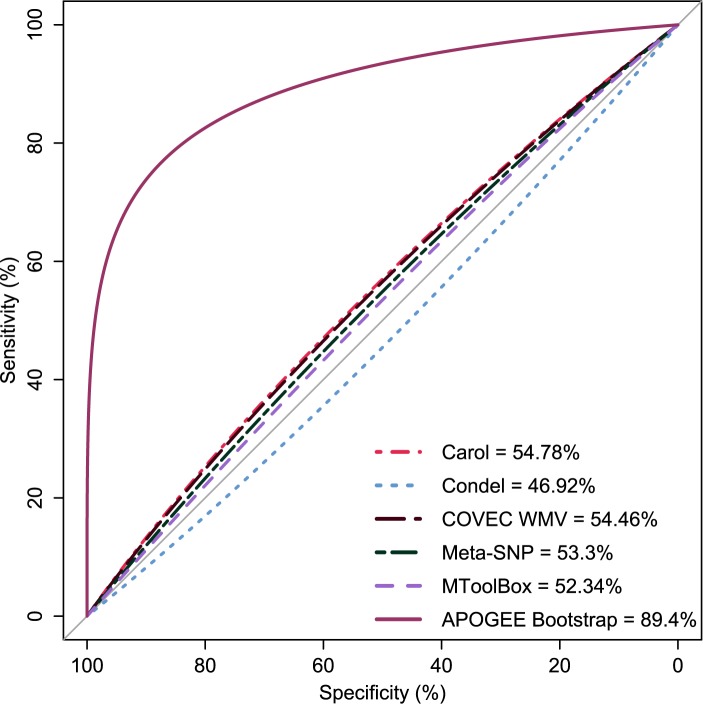
Meta-predictors performance comparisons by receiver operating characteristic curves.

APOGEE outperformed all by achieving the best sensitivity, accuracy, precision, FDR, MCC and MCR rates and the second highest specificity value (after FatHmm) (Table 1 and Fig 1).

The risk of overfitting was checked against two additional test sets, not overlapping with the training set (S2 Text). One was made of 153 variants appearing in the latest releases of dbSNP and MITOMAP, at the time of this writing. The classification rates of APOGEE resulted at least as high as those reported in Table 1 (cf. Table 2). The latter independent test set was made of 48 unbiased variants, on which APOGEE obtained the performance records reported in Table 3, which are in line with those previously shown.

**Table 2:** Performance evaluation calculated on 153 known and unbiased mitochondrial non-synonymous variants.

	*N*	*P*	*Total*
***N***	115 TN = 83.33%	23 FP = 16.17%	**138**
***P***	5 FN = 33.33%	10 TP = 66.67%	**15**
***Total***	**120**	**33**	**153**

**Table 3:** Performance evaluation calculated on 48 known and unbiased mitochondrial non-synonymous variants.

	*N*	*P*	*Total*
***N***	30 TN = 76.92%	9 FP = 23.08%	**39**
***P***	2 FN = 22.22%	7 TP = 77.78%	**9**
***Total***	**32**	**16**	**48**

## Discussion

Software predictors of the harmfulness of genomic variations were shown to be incongruent [2]. The major cause of incongruence was ascribed to two types of *circularity issues* affecting both training and test datasets used by data mining-based predictors [13]. Type 1 refers to the accidental, even if frequent, partial overlap between the training and test datasets. Type 2 consists in deeming all variants of some genes as pathogenic or neutral, for the mere fact of falling within a functionally critical gene. The consequence of that was a strong bias towards pathogenic or neutral predictions for them, and thus an increasingly low prediction sensitivity. Unfortunately, being the mitochondrial genome very small and each gene relatively little affected by mutations, both type 1 and 2 problems are unavoidable, even if reducible. Type 2 circularity problem has limited impact on the mtDNA, since for all 13 protein-coding genes, both true neutral and true harmful missense substitutions are reported, with a proportion of deleterious variants ranging from 15% to 35%. Considering the low number of genes and the disproportion between harmful and neutral mutations, the type 2 problem has a globally reduced effect on the predictions, and will tend to disappear with new findings. In principle, type I problem might be significantly cut down by finely curating the training sets.

The strategy implemented in APOGEE, which made it the best performer, consisted in adopting a transparent machine-learning algorithm that yielded a number of decision rules taken on larger and finely curated training sets. The LMT classifier was not claimed here to perform better than any other machine learning algorithms by far, but to perfectly fit the need for a dichotomous classifier that delivers a probability for a variant to be pathogenic, together with the rule according to which the decision is taken. The extra and decisive step consisted in tackling the longstanding problem of artifacts and misclassified variants of training sets by (i) discarding variants if originated from alignment errors; (ii) flipping the outcomes of the predictions (pathogenic and neutral) when new phenotypes or clinical evidences become available; (iii) removing false variants corresponding to alleles excluded from multi-allelic sites after periodic dbSNP update. Some variants of public datasets were indeed poorly annotated or simply artifacts. We bumped against a number of these along the previous three versions of the training sets of APOGEE. Several variants from a preceding version of the training set were updated in the subsequent, either because new experimental evidences reverted their estimated pathological effects, or because they were finally associated with any disease or in case of multiallelic sites. In particular, a few multiallelic sites were reassessed since only one allele was actually validated, with the others being deemed artifacts. Other variants were completely removed since they were found not to map to any assembled mitochondrial sequence present in dbSNP. 432 core variants were shared among all three datasets, 230 of which were considered functionally neutral and 202 pathogenic. 215 in 230 were observed to be actually neutral in all three training sets. 181 in 202 were unanimously considered deleterious.

This preprocessing step contributed to obtain a finely curated and larger training set. Most classifiers were indeed trained on a handful of known variations, as for example, MToolBox DS, which was built on the 53 damaging missense variants available from the Humsavar dataset (Table 3 in [14]). On the contrary, APOGEE was trained on a total of 223 deleterious variants.

### Future directions

The proportion of neutral and pathogenic amino acid changing variants that occur in a gene sequence mainly depends on the mutational pressure, genetic drift and both purifying and adaptive selection. These evolutionary mechanisms are generally considered gene-specific, thus making difficult the identification of potential deleterious mutations without any knowledge of the gene-specific level of tolerance to mutations. Therefore, taking into account the evolutionary measure of a gene or of a gene family, meant as the ratio between the non-synonymous and synonymous substitution rates, as calculated in a set of aligned orthologous sequences [15], could dramatically increase the sensitivity of the predictors. A beneficial effect might also be conferred by the RVIS index [16], which determines which nuclear genes are more intolerant to missense mutations. These two indices might provide useful insights in the understanding of the different evolutionary dynamics of genes and will be integrated in future releases of APOGEE.

The functional role of each mutant residue is greatly influenced by the co-inherited missense variants within the very same protein or within structurally/functionally associated proteins. This “coevolutionary issue” has been poorly investigated so far, although it is well known that human pathogenic mutations can also be present within other species, without no deleterious effects, because they are probably compensated by co-inherited intra- or inter- gene mutations. Currently, a novel computational strategy has been developed in order to identify human pathogenic mutations that are *compensated* in extra-specific genomes (Compensated Pathogenic Deviations), i.e., their damaging effects are counterbalanced by other fixed mutations that are absent in humans [17]. A relevant proportion (3–10%) of human damaging missense mutations has been identified in mammal and vertebrate protein alignments, indicating that compensatory mechanisms exist (sequences are assumed to derive from healthy animal organisms) at different evolutionary ages [17].

The identification of coevolving residue pairs is impeded, at any rate, by the paucity of appropriate experimental data. Knowledge of the ternary and quaternary structures of mitochondrial and nuclear OXPHOS proteins could contribute to resolve the inconsistencies among computational pathogenicity predictions and diseases association [18]. This aspect will also be taken into consideration in the next releases of APOGEE.

## Materials and methods

### Data sources of training sets of known variants

Variants with known functional effects on mitochondrial proteins were harvested from MITOMAP [8] (accessed July 2015) and dbSNP 144 [19]. In total, we collected 864 non-synonymous mutations, 228 of which were tagged as “confirmed” or “reported” in MITOMAP. 223 were already linked to known mitochondrial genetic disorders (i.e., Leber optic neuropathy (LHON), mitochondrial encephalomyopathy, lactic acidosis, stroke-like episodes, maternally inherited deafness or aminoglycoside-induced deafness), or complex diseases such as Alzheimer or cancer. 30 on 228 were confirmed to be pathogenic amino acid changing variants and most of them resulted to cause LHON. On the other hand, 5 out of 228 (8741:T>G, 8795:A>G, 9055:G>A, 8414:C>T, 3745:G>A) were reported as non-pathogenic, thus exhibiting a likely protective or compensatory effect on the carrier subjects. The remaining 699 variants were retrieved from Ncbi dbSNP 144 through the Ncbi Variation Reporter tool (http://www.ncbi.nlm.nih.gov/variation/tools/reporter). Variants with no reported pathological consequences in dbSNP and no overlap with MITOMAP were considered harmless. In detail, 63 of the 699 dbSNP variants were classified as pathogenic, being these present in MITOMAP. The remaining variants were considered neutral (cf. Table 4). Hence, the entire variant set consisted of 223 pathogenic (MITOMAP), 5 non-pathogenic (MITOMAP) and 636 (non-overlapping dbSNP) neutral variations (cf. S1 Table).

**Table 4:** Known variants grouped by mitochondrial gene symbol and OXPHOS complex.

*Complex*	*Gene*	*# variants*	*# pathogenic* *variants (%)*	*# described in* *MITOMAP (%)*	*# reported in dbSNP (%)*
*I*	ATP6	78	19 (24.4%)	22 (28.2%)	62 (79.5%)
	ATP8	18	6 (33.3%)	7 (38.9%)	15 (83.3%)
*IV*	COX1	81	25 (30.9%)	25 (30.9%)	62 (76.5%)
	COX2	50	14 (28%)	14 (28%)	37 (74%)
	COX3	59	10 (16.9%)	10 (16.9%)	49 (83.1%)
III	CYB	98	33 (33.7%)	33 (33.7%)	79 (80.6%)
*V*	ND1	103	37 (35.9%)	38 (36.9%)	75 (72.8%)
	ND2	60	12 (20%)	12 (20%)	49 (81.7%)
	ND3	26	6 (23.1%)	6 (23.1%)	22 (84.6%)
	ND4	74	12 (16.2%)	12 (12%)	68 (91.9%)
	ND4L	25	3 (12%)	3 (12%)	23 (92%)
	ND5	140	28 (20%)	28 (20%)	117 (83.6%)
	ND6	52	18 (34.6%)	18 (34.6%)	41 (78.8%)

Our APOGEE classifier was trained on these datasets, as specified below, and tested also on two non-overlapping datasets. In particular, we have put together a set of 153 new functional variants, which came from the latest releases of MITOMAP (accessed in January 2017) and dbSNP (ver. 147), and additional 48 variants obtained from VariBench [13] (web-site: http://structure.bmc.lu.se/VariBench/GrimmDatasets.php). We made sure that these variants were not included in our original training sets.

### Assembled predictors in MitImpact

Assessments of pathogenicity were computed by and collected from a number of predictors (Table 5), provided that these could process batch queries and accepted mitochondrial protein-coding gene symbols in input [20]. We used EFIN with standard parameters, after training it on SwissProt (SP) [21] and HumDiv (HD) [22] datasets. Similarly, we queried CADD 1.3 [23] and obtained two scores, the original and the phred-scaled scores. Being numeric, we dichotomized the phred scores and classified the variants that exceeded the threshold of 12 as harmful, as suggested by the authors. Variants were submitted to CADD in VCF-like data format. We further retrieved predictions from CRAVAT [24], both for mendelian (VEST) [25] and cancer (CHASM) [26] diseases. Input variants were specified as Ensembl Transcript IDs and amino acid substitutions, using the one-letter encoding. It responded to our query with pairs of p-values and FDRs, one for each input variant. If a prediction was significant, i.e., p-value <0.05 and FDR <0.2, we labeled the corresponding variant as pathogenic (in case of VEST) or driver (in case of CHASM). We applied the *weighted* version of the FatHmm prediction algorithm [27] to a list of Uniprot accession numbers and amino acid substitutions and obtained functional scores and categorical predictions for them. Likewise, we queried the Meta-SNP server [28], but submitting the fasta sequences of the OXPHOS proteins and the corresponding lists of amino acid mutations. It returned categorical predictions and scores for PhD-SNP, SIFT, SNAP and PANTHER [29–32].

**Table 5:** List of assembled predictors and annotations in MitImpact.

*Features*	*Tools*
*Pathogenicity predictors*	PolyPhen2, SIFT, FatHmm, PROVEAN, MutationAssessor, EFIN, *CADD*, *FatHmm_w*, *VEST*, *PANTHER*, *PhD-SNP*, *SNAP*
*Meta predictions*	CAROL, Condel, *COVEC*, *Meta-SNP*, *MtoolBox Disease Score*
*Cancer-specific predictions*	*PolyPhen2 transf*, *SIFT transf*, *MutationAssessor transf*, *CHASM*
*Variant annotations*	dbSNP 144, COSMIC 68, MITOMAP July 2015
*Evolutionary indexes*	PhyloP100V, PhastCons100V, SiteVar, *MISTIC coevo*

MitImpact accounted also for the MtoolBox Disease Scores. We set the pathogenicity threshold to 0.4311, as described in [14] (details in S1 File), and considered harmful all variants exceeding it. We additionally included the COVEC 0.4 scores [33]. We run the COVEC Weighted Majority Rule algorithm and obtained a numerical score for each variant, based on a consensus of the predictions of SIFT, PolyPhen2 and MutationAssessor. A pathological status was associated to a variant if its COVEC score was positive. Similarly, we computed the Transformed Functional Impact for Cancer (TransFIC) score [34], by providing TransFIC with the SIFT, PolyPhen2 [22] and MutationAssessor [35] scores, which were already stored in the former release of MitImpact [36]. TransFIC normalized these scores on a *baseline tolerance of genes*, which corresponded to the level of tolerance of germline variants occurring in genes with dissimilar functions. Functional similarity was assessed on the Gene Ontology Biological Process annotation (gosbp). The tool yielded a tripartite categorical classification for each variant given in input, along with the transformed scores.

MitImpact took into consideration also cancer-related information, taken from COSMIC 68 [37]. COSMIC IDs and information on the tumor type, number of examined tumor samples and mutation frequency for the matching variants were included. Moreover, the conservation indices PhyloP100V and PhastCons100V [38] were calculated for all the mitochondrial genomic positions that cause missense substitutions by using the UCSC Gene Tables gateway. We additionally included information on protein coevolution through the MISTIC [39] webserver, a tool that predicts coevolving sites within mitochondrial protein sequence alignments. We retrieved protein alignments from the Ncbi Organelle Genome resource, restricting the study to Mammals (about 670 species-specific sequences for each gene) and using the human protein sequences as reference. We then computed the matrix of Mutual Information (MI) scores (MI Z-scores), which contains the scores of all the possible amino acid pairs, and then selected only the pairs with scores > 6.5, since these are suggested by the authors to be coevolving pairs of amino acids. Then, we calculated the frequency of the coevolving amino acids and the mean MI Z-score for each amino acid site.

These scores were computed for all 24,189 non-synonymous amino acid changes potentially affecting the human mitochondrial DNA and made freely available, as a flat-file with variants as rows and scores as columns, from MitImpact. Variants were grouped in training and test sets, as for the previous section, and used to build and verify the APOGEE classifier.

### The APOGEE classifier

The predictions of the abovementioned tools were used to feed APOGEE (pAthogenicity Prediction thrOugh loGistic modEl trEe). Its operating logic bases on the classification model of the Logistic Model Tree (LMT). The choice of yet another meta-predictor was driven by our intent to offer a transparent classifier, finely tuned on mitochondrial variants and that gives reproducible and easy-to-understand results. LMT combines the logistic regression models with tree induction resulting in a single tree. It uniquely provides the user with *decision rules* that allow, easily, classifying unknown variants as neutral or harmful. Moreover, LMT has the advantage of providing explicit class probability estimates and, thus, of helping the user to intuitively grasp the actual uncertainty behind any evaluation of pathogenicity.

It builds a standard decision tree structure with logistic regression functions at the leaves. Each leaf may not contain the same function, since variables are independently selected to maximize the discrimination between neutral and pathogenic mutations. The tree-induction procedure proceeds in a *top-down* fashion. It recursively splits the instances (variations) space and stops when the inferred subdivisions are reasonably “pure”, in the sense that they contain observations with mostly identical class labels (pathogenic or neutral). In a standard decision tree framework, a region is labeled with the majority class of the observations in that region.

Formally, we inferred an unknown function *f*, which can map the predictor variables *X*_s_ to the class label *Y*:

$$Y=f(X_{1},\ldots ,X_{p}),$$

where *X*_s_ were the pathogenicity scores, while the response variable *Y* was the target class. The function *f*(⋅) was directly inferred from real data, which consisted of a set of *n* variations, carrying their pathogenicity scores *p* along with their classes (or labels) *y* of belonging.

By denoting the *n* × *p* data matrix (without labels y) with bold **X** and the **p**-dimensional vector of annotation scores for a single mutation with **x**, we modeled the posterior class probabilities *P*(*Y*|*X*) using a sigmoid function. For a two-class classification problem, for which we specify the labels of *Y* as *y* = ±1 (with 1 for neutral mutations and -1 for pathological mutations):

$$P(Y=y|x,w)=\frac{1}{1+e^{-yw^{T}x}}$$

or, equivalently:

$$P(Y=1|x,w)=\frac{e^{w^{T}x}}{1+e^{w^{T}x}}\;\mathrm{or}\;P(Y=-1|x,w)=\frac{1}{1+e^{w^{T}x}}.$$

Here, **w** is the unknown vector of *p* weights associated with each predictor. In order to compute the model for each class, we estimated these weights. This was achieved through the minimization of the following logistic cost function:

$$\hat{w}=\underset{w}{argmin}\sum_{i=1}^{n}log(1+e^{-y_{i}w^{T}x_{i}}).$$

Once the weights were computed, the final regression model for each class was determined through a *LogitBoost* algorithm, which selected the final predictors (*x*_*i*_) to be included in the model. Therefore, we obtained that:

$$P(Y=y_{j}|x,\hat{w})=\frac{e^{F_{j}(x)}}{1+\sum_{k=1}^{J}e^{F_{k}(x)}},\sum_{k=1}^{J}F_{k}(x)=0,J=2$$

where *F*_*k*_(*x*) was the estimated logistic function of the *k*^th^ class. The class labels of the mutations were assigned by the following formula:

$$y^{*}=\underset{y}{argmax}\;P(Y=y|x,\hat{w}).$$

As mentioned earlier, the tree structure gives a disjoint subdivision of the whole instance space *S*, spanned by all pathogenicity scores (or predictors) that are present in the data, into regions *S*_*t*_. Every region was represented by a leaf in the tree:

$$S=t\in TS_{t},\;S_{t}\cap S_{t^{\prime }}=\emptyset \;\text{for}\;t\neq t^{\prime }$$

A logistic regression function *f*_*t*_ was associated to each leaf *t* ∈ *T*, which included a subset *V*_*t*_ ⊆ *V* of all pathogenicity scores present in the data and that modeled the class membership probabilities as *P*(*Y* = *y*|***x***,***w***). The weight estimates were zero when the predictor did not contribute to the model. Generalizing the whole LMT model:

$$f(x)=\sum_{t\in T}f_{t}(x)∙I(x\in S_{t})$$

where *I*(*x* ∈ *S*_*t*_) is a variable indicator that equals 1 if the observation *x* belongs to the region *S*_*t*_ or zero, otherwise.

Under or over-estimation of the prediction capability of APOGEE would be possible if considering only one run of the algorithm, in a similar setting with unbalanced class sizes (i.e. 223 pathogenic vs 641 benign mutations). This dimensional bias was tackled by the implementation of a bootstrap strategy that, by definition, is based on randomly drawing a sample with replacement from the observed sample of size n = 223 for pathogenic variants and n = 641 for tolerated variants. The random sampling was repeated 100 times, resulting in 100-bootstrap samples. For any given draw, approximately one-third of observations were not selected and served as test set (out-of-bag (OOB) test set). Subsequently, the LMT was applied to each of the 100-bootstrap samples and a prediction error assessed using the corresponding 100 test sets, namely those observations not included in the training set due to sampling with replacement. This measure of prediction error is referred to as leave-one-out bootstrap estimate. [40]. Thus, the fact of sampling the 70% of all pathogenic variants and the same number of the neutral variants implied that the expected frequencies of inclusion of both types of variants were 50% and 22%, respectively. In brief, for 100 iterations, we run this algorithm:

**Step 1:** Sampling the training set, as described above;
**Step 2:** Estimating the LMT;
**Step 3:** Predicting the pathogenicity of all the mutations stored in the database.

Each iteration gave an estimate of the pathogenicity of the variants in the OOB set. A variant was deemed harmful if the mean of the probabilities of being harmful, calculated for all iterations in which it was included in the OOB, resulted > 0.5. Compared to an individual run, bootstrap replaces the classification rules of an LMT model with the probability of being harmful. The classifier was implemented in R, by using the R package Rweka [41] [42].

### Availability

APOGEE is freely available in MitImpact [36] at http://mitimpact.css-mendel.it/.

## Supporting information

S1 Text Details on APOGEE and classification performance comparison. (DOCX)

S2 Text Unbiased, non-overlapping, test sets of variants from MITOMAP and VariBench. (XLSX)

S1 Table Training set of 864 variants with known pathogenic impact. (XLSX)
