# Supplementary material for: High-confidence assessment of functional impact of human mitochondrial non-synonymous genome variations by APOGEE
Source: PLoS Comput Biol. 2017 Jun 22;13(6):e1005628. doi: 10.1371/journal.pcbi.1005628 (PMC5501658; doi:10.1371/journal.pcbi.1005628)
Supplement: S1 Text — (DOCX) [file pcbi.1005628.s001.docx]

Supplementary File

Table of Contents

[Details of the methods for pathogenicity estimation 2](#_Toc477600448)

[Distribution of the assembled pathogenicity scores 3](#_Toc477600449)

[Pairwise agreement assessment between categorical pathogenicity predictions 11](#_Toc477600450)

[Details of APOGEE logistic model trees 12](#_Toc477600451)

[Comparison of datasets of known pathogenic variants 12](#_Toc477600452)

[Availability and query-ability of data 13](#_Toc477600453)

# Details of the methods for pathogenicity estimation

Conditions for inclusion in this study for a predictor were:

- Ability to recognize mitochondrial gene sequences or accession numbers (e.g. UNIPROT or Ensembl Protein IDs);
- To be implemented as an easy-to-use web interface or as an easy-to-configure standalone package;
- Ability to process large numbers of variants simultaneously and in a reasonable time;
- Not present in the first release of the database, unless this was a new algorithm or implementation (e.g. FatHmm weighted (original version) vs. FatHmm unweighted (latest version)).

Some predictions were obtained through the web-interfaces of PolyPhen2.2.2, SIFT 5.0.3, FatHmm 2.2, PROVEAN 1.1.3 and MutationAssessor2 (see ^1^ for details). EFIN, CADD 1.3, FatHmm 2.3 weighted, CRAVAT-VEST (November 2012 release) web-servers were queried in June-July 2015. PANTHER, PhD-SNP and SNAP results were obtained through Meta-SNP (accessed in June-July 2015). For what concerns the meta-predictors, CAROL, Condel (2014 version) and COVEC ver. 0.4 were run locally with the following command lines:

“R CMD BATCH '--args Carol_input.txt' CAROL_script.r CAROL_results.Rout"

“perl was_SPM.pl /condel/condel/config Condel_input.txt > Condel_output.txt”

“perl wv.pl Covec_input.txt Covec_output.txt”

*Carol-, Condel-* and *Covec-input* are data tables containing PolyPhen2/SIFT scores (CAROL and Condel) or PolyPhen2/SIFT/MutationAssessor categorical predictions (COVEC weighted majority rule).

TransFIC (cancer-related pathogenicity prediction) was run locally with the following command line:

“perl transf_scores.pl gobp Transfic_input.txt > Transfic_out.txt”,

where the “gobp” argument indicates the use of the “Gene Ontology Biological Process Partition” as a reference; the “Transfic_input” table provides the original SIFT/PolyPhen2/MutationAssessor scores.

The Disease Score of MToolBox (MToolBox DS) was obtained from the file “patho_table.txt”, available from the MToolBox web site, by matching the genomic positions of the missense variants stored into the MitImpact and MToolBox databases. Categorical predictions (*deleterious*, *neutral*) were assigned to variants according to whether their DSs were greater or not than 0.4311, as suggested by the authors of MToolBox^2^.

CHASM scores were obtained by querying the CRAVAT webserver. Additionally, it provided also VEST predictions.

Pathogenicity predictors can be accessed at the following web URLs:

| PolyPhen2 | http://genetics.bwh.harvard.edu/pph2/ |
| --- | --- |
| SIFT | http://sift.bii.a-star.edu.sg/index.html |
| FatHmm | http://fathmm.biocompute.org.uk/ |
| PROVEAN | http://provean.jcvi.org/ |
| MutationAssessor | http://mutationassessor.org/ |
| EFIN | http://paed.hku.hk/efin/ |
| CADD | http://cadd.gs.washington.edu/ |
| VEST | http://www.cravat.us/ |
| Meta-SNP | http://snps.biofold.org/meta-snp/index.html |
| COVEC | http://sourceforge.net/projects/covec/files/ |
| CAROL | http://www.sanger.ac.uk/science/tools/carol |
| Condel | http://bg.upf.edu/fannsdb/ |
| TransFIC | http://bg.upf.edu/fannsdb/ |
| CHASM | http://www.cravat.us/ |
| MToolBox DS | https://github.com/mitoNGS/MToolBox/blob/master/MToolBox/data/patho_table.txt |

# Distribution of the assembled pathogenicity scores

For all the possible 24,189 mtDNA non-synonymous amino acid changes, as well as for 2 subsets of experimentally validated pathogenic and neutral variants (n=223 and n=641), we computed, plotted and reported the distributions of their pathogenicity scores.

PolyPhen2, which was trained with the HDiv dataset, generally over-estimated the pathogenicity of variants and thus predicted most variants as probably or possibly damaging. Although the median values of the scores of the putative neutral and pathogenic variants were significantly different (Wilcoxon rank sum test, p-value= 3.84E-4), the scores appeared interspersed, with many observed pathogenic mutations exhibiting very low scores, thus being false negative. Conversely, the distribution of the SIFT scores tended mostly to neutrality. The median score for the pathogenic set of variants was correctly lower than that of the set of neutral variants (Wilcoxon rank sum test, p-value=2.34E-3), even if it was generally greater than the SIFT’s pathogenicity threshold that is fixed to 0.05. For this reason, many real pathogenic variants were classified as neutral. PROVEAN [14] scores ranged from -15 (prevalently pathogenic) to 10 (prevalently neutral), with the pathogenic subset having generally lower scores (Wilcoxon rank sum test, p-value=4.56E-3). The Functional Impact (FI) scores produced by MutationAssessor ranged from -5 to 6 (from neutral to high functional impact), with the neutral subset tending to lower values (variants categorized as neutral or low functional impact) with respect to the pathogenic subset (Wilcoxon rank sum test, p-value=2.58E-6). Regarding to the two implementations of EFIN, scores were skewed, when training with the HumDiv dataset (EFIN-HD) compared to the SwissProt-trained version (EFIN-SP). Scores were generally higher for the latter implementation. EFIN-SP and EFIN-HD pathogenic variants exhibited lower scores than the neutral subsets (Wilcoxon rank sum test, p-value=1.27E-6 and p-value=5.58E-3, respectively). Phred-scaled CADD scores generally ranged from 0 to 15, with an outlier variant of the gene ND1 with a score of 18.43 (3838:T>C). Distributions of scores were quite overlapping, with deleterious mutations exhibiting higher scores (Wilcoxon rank sum test, p-value=3.72E-3). Scores of PANTHER, PhD-SNP e SNAP ranged from 0 (putative neutrality) to 1 (putative pathogenicity), with pathogenic variants having generally higher values for PhD-SNP e SNAP (Wilcoxon rank sum test, p-value=2.53E-4 and p-value=2.38E-5) and similar values for PANTHER (Wilcoxon rank sum test, p-value=0.08), compared to neutral variants. The scores provided by the unweighted version of FatHmm ranged from -3 (putative deleterious) to 6 (functional neutrality), with the median scores of both classes being very close (Wilcoxon rank sum test, p-value=0.605). The weighted version, instead, which recalibrates the predictions on the protein functions, provided scores in the intervals (-15, 5), with known pathogenic variants (223 mutations) exhibiting significantly lower scores than neutral variants (p-value=7.33E-4). VEST scores, which were accompanied with p-values and FDRs, were filtered and considered only if p-values < 0.05. Of 3,145 in 24,189 putative pathogenic amino acid substitutions, 33 were known to have a functional effect, while 59 were surely neutral. FDR values were generally pretty high for most variants, with only 1,356 in 24,189 having a FDR of at least 0.4.

The scores obtained with the meta-predictors CAROL, Condel and Meta-SNP ranged from 0 (no functional impact) to 1 (high functional impact). Generally, CAROL was biased against neutrality and, consequently, the pathogenic variants exhibited significantly higher scores than neutral variants (Wilcoxon rank sum test, p-value = 8.39E-5). Conversely, the distribution of Condel scores was generally shifted towards neutrality, with many known harmful variants exhibiting, erroneously, lower scores than true neutrals (Wilcoxon rank sum test, p-value=1.31E-4). Meta-SNP scores were biased towards pathogenicity. The median values of the harmful and neutral variant sets were significantly different (Wilcoxon rank sum test, p-value=5.28E-4). The MToolBox Disease Scores (DSs) were significantly higher for pathogenic variants than for neutral (Wilcoxon rank sum test, p-value=6.28E-5). Regarding the COVEC Weighted Majority Vote scores, we found that pathogenic variants received, on average, a higher score than the neutral set (p-value=1.15E-4).

Differently from all the above meta-predictors, APOGEE returns the probability for a variant of being harmful. We examined the distribution of its scores, when built on three different training sets of increasing sizes (cf. Influence of the training set quality on the APOGEE prediction performance, for details). With the first version (TDNov2014), variants globally displayed probabilities around 0.5. Median values of the probabilities of being damaging or neutral differed significantly (Wilcoxon rank sum test, p-value=7.82E-9). When training APOGEE with the second set of known variants (TDJan2015), probabilities shifted towards neutrality, but with several outliers. A great proportion (around 40%) of known damaging variants exhibited a probability of being harmful >0.5, and these were generally higher than the probabilities of true neutrals (Wilcoxon rank sum test, p-value=6.67E-13). Finally, when we trained APOGEE with TDJul2015, we observed that the median probability of being pathogenic was lower than that calculated with TDJan2015 (i.e., variants were more prone to be classified as neutral, even if the median score of the pathogenic variants resulted significantly higher than that of the neutral variants, Wilcoxon rank sum test, p-value=2.87E-11). APOGEE prediction capability was boosted with a bootstrap strategy (Table 1), which reduced the overall misclassification rates from 22.67% (TDJan2015) to 15.97% (APOGEE Bootstrap). As a side effect, the introduction of the bootstrap strategy caused a slight increase of the false positive rate.

In summary, for each predictor (PolyPhen2, SIFT, FatHmm, FatHmm_W, PROVEAN, MutationAssessor, EFIN (SwissProt and HumDiv models), CADD, PANTHER, PhD-SNP and SNAP) and meta-predictor (CAROL, Condel, Meta-SNP, MToolBox and APOGEE), we plotted the distribution of scores as boxplots for the entire MitImpact dataset (i.e., 24.189 variants) and for the 641 known neutral and 223 pathogenic variants.


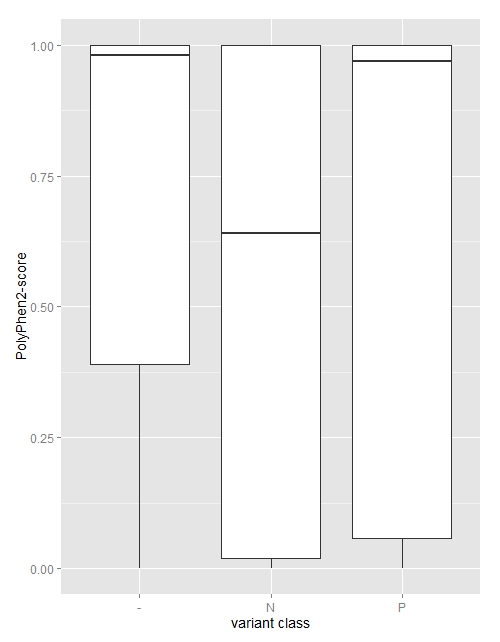

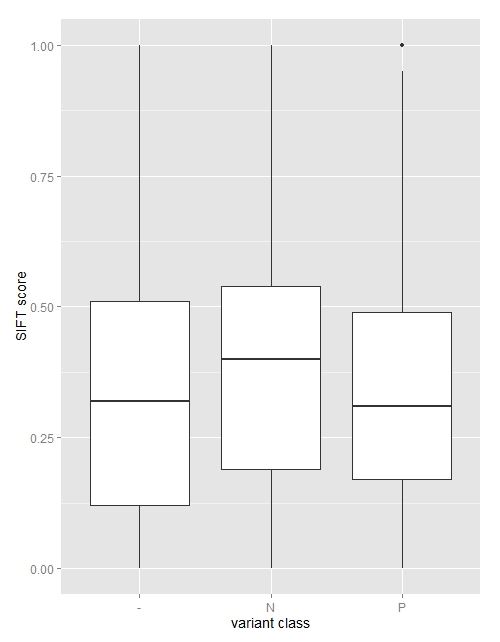


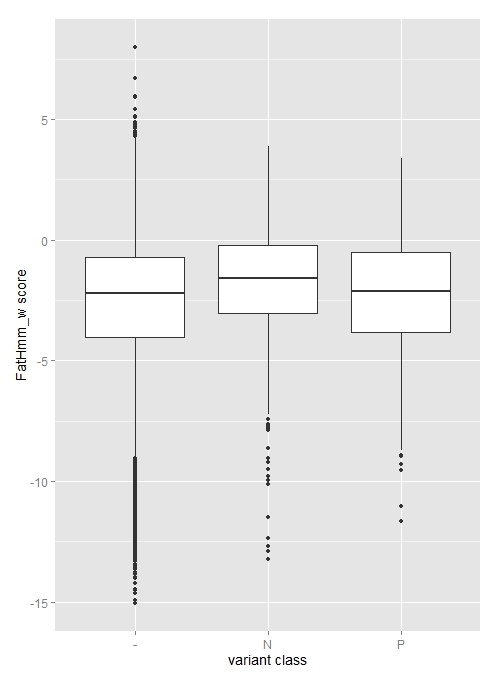
**
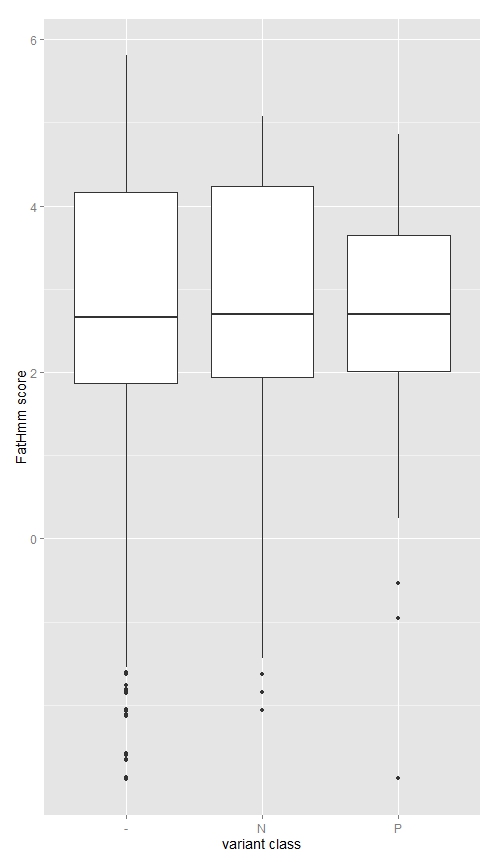
**

**
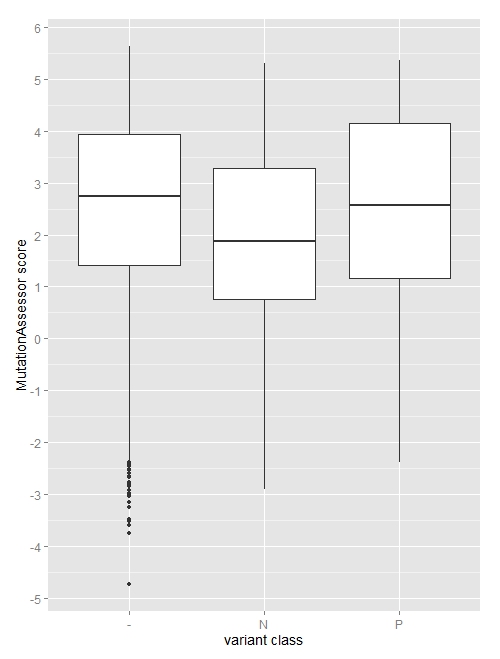

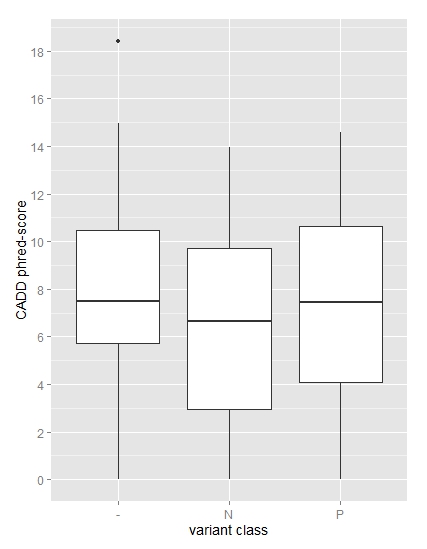
**

**
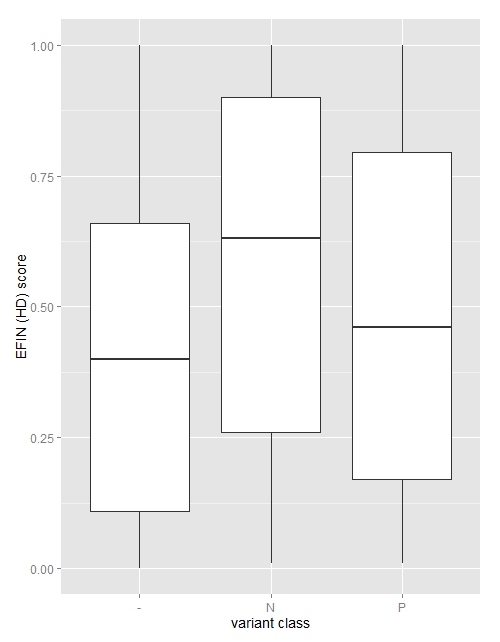

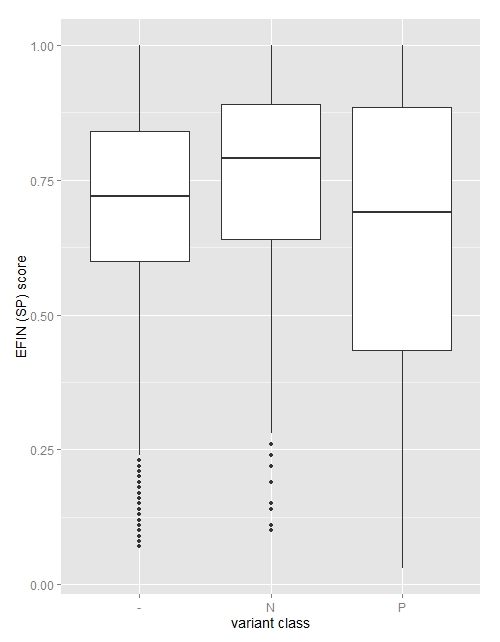
**

**
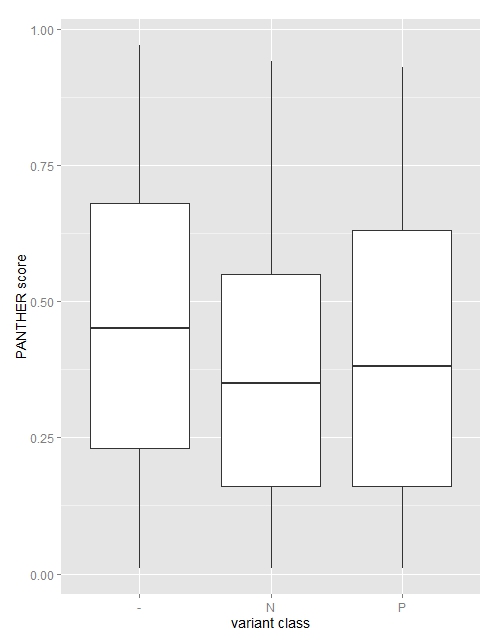
**
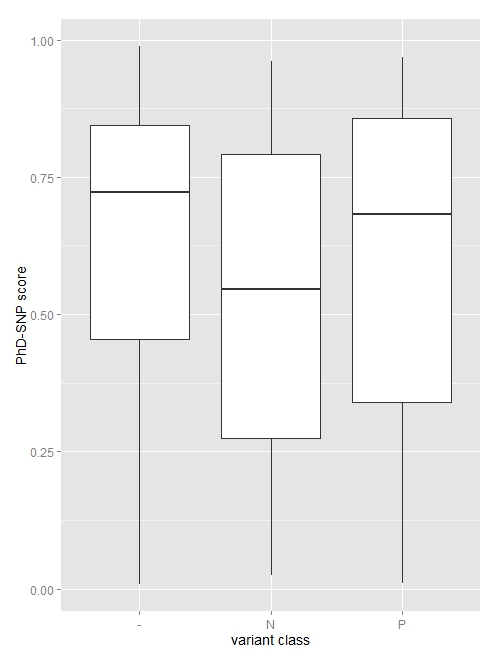


**
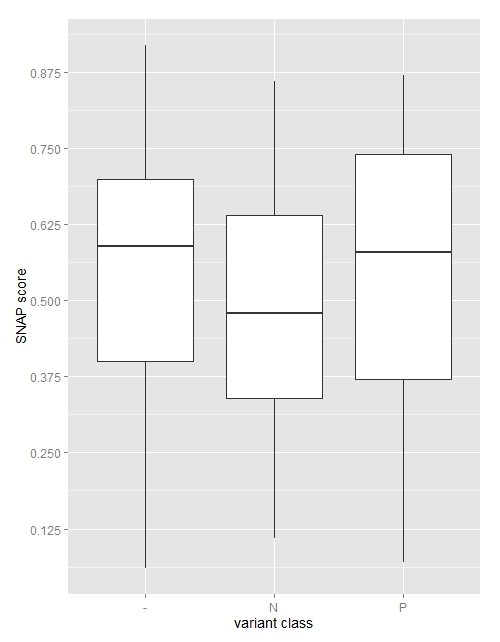

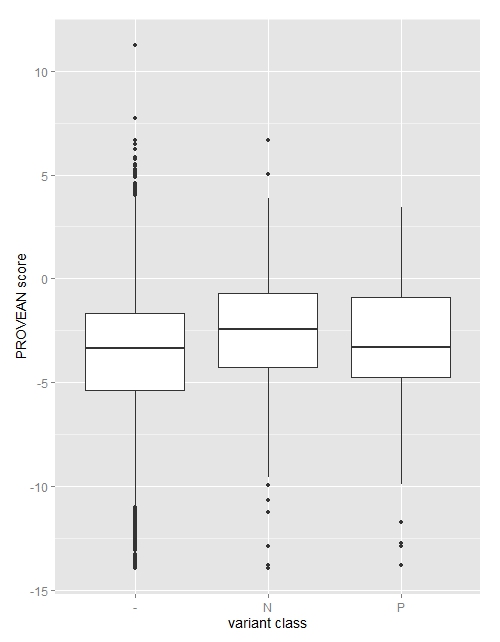
**

VEST plots: p-values associated to the prediction scores vs. False Discovery Rates (FDRs), for the whole dataset. Note that no categorical predictions were provided by this method.

**
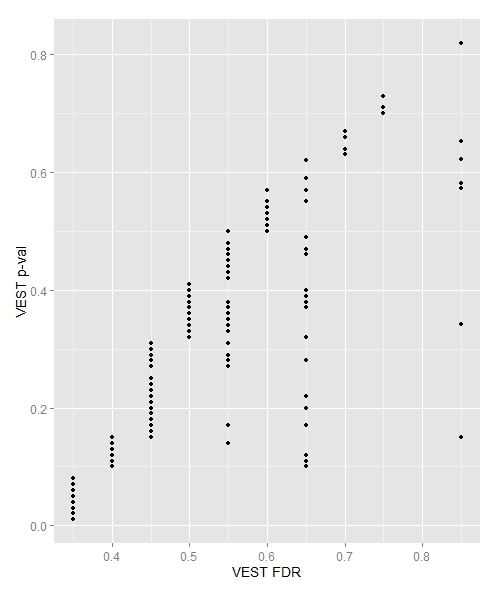
**

VEST plots: p-values associated to the prediction scores vs. FDRs of known neutral variants.


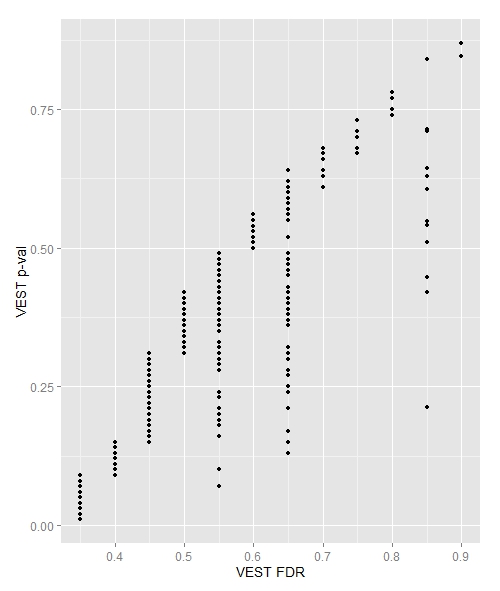


VEST plots: p-values associated to the prediction scores vs. FDRs of known pathogenic variants.


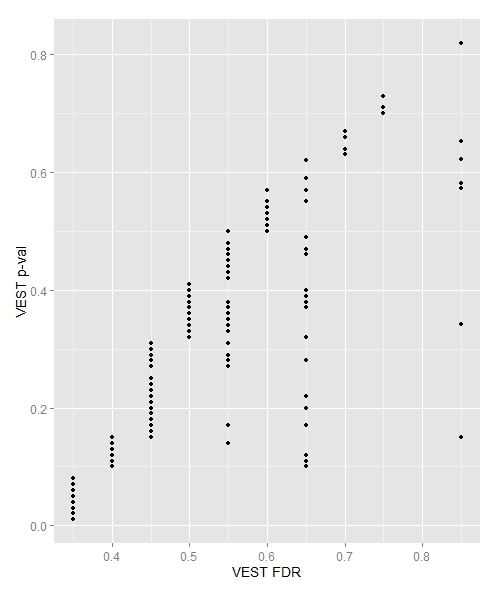


Boxplots of scores of the meta-predictors: CAROL, Condel, Meta-SNP, APOGEE TDNov2014, APOGEE TDJan2015, APOGEE TDJul2015 and MToolBox. ‘-‘ class includes all unknown mitochondrial variants; ‘N’ and ‘P’ classes contain all known neutral and pathogenic variants.

**
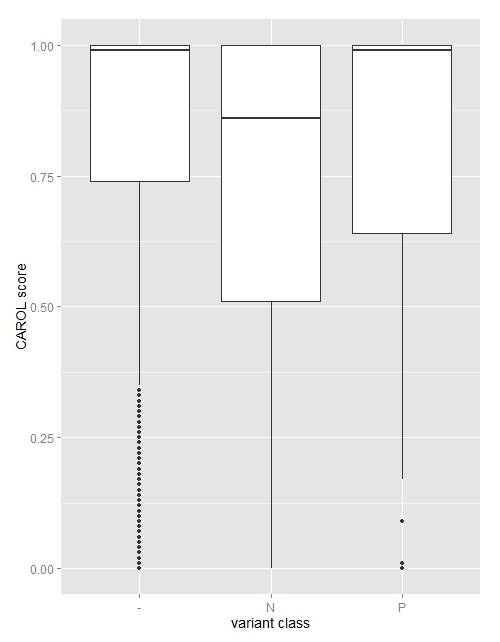
**

**
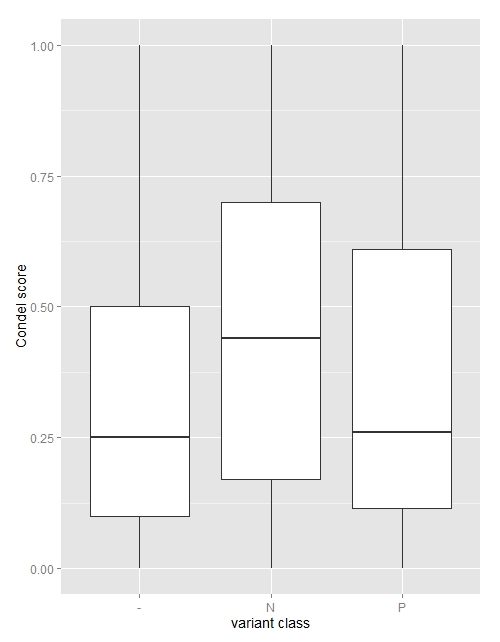

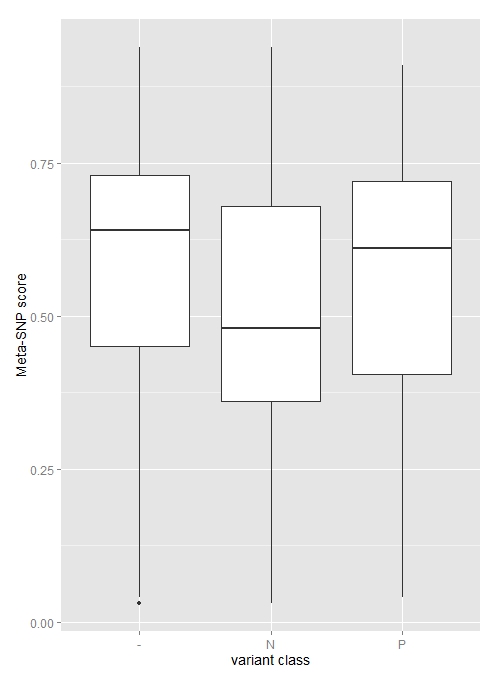
**

**
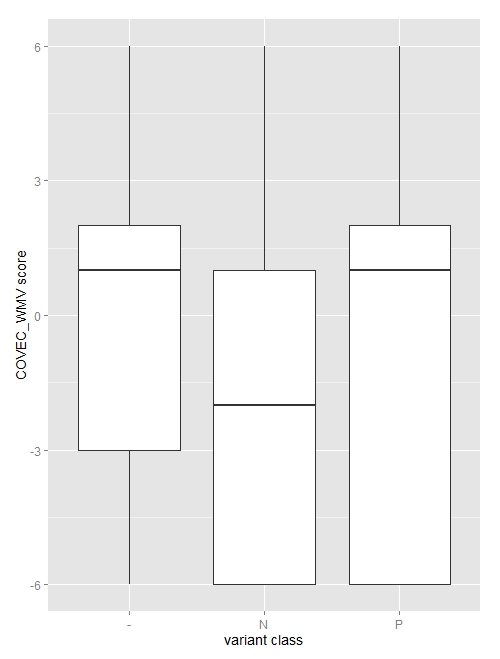

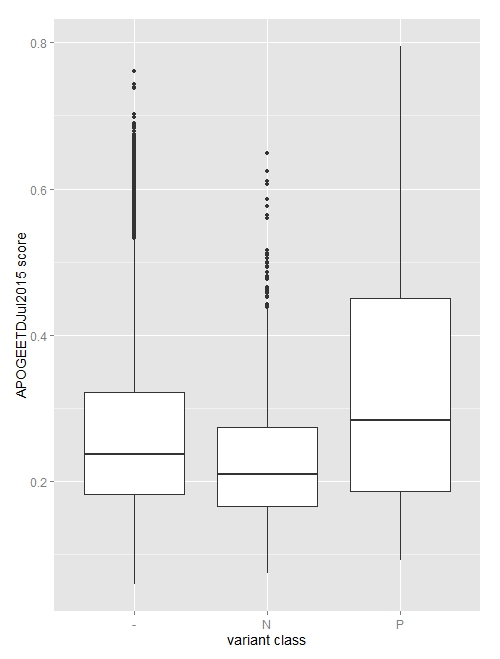
**

**
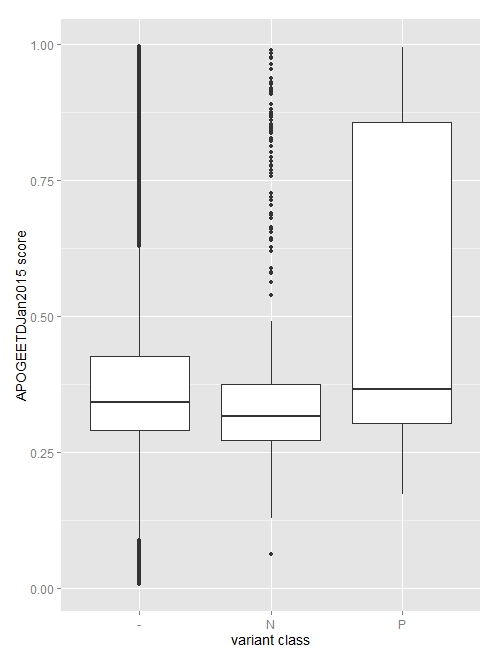
**
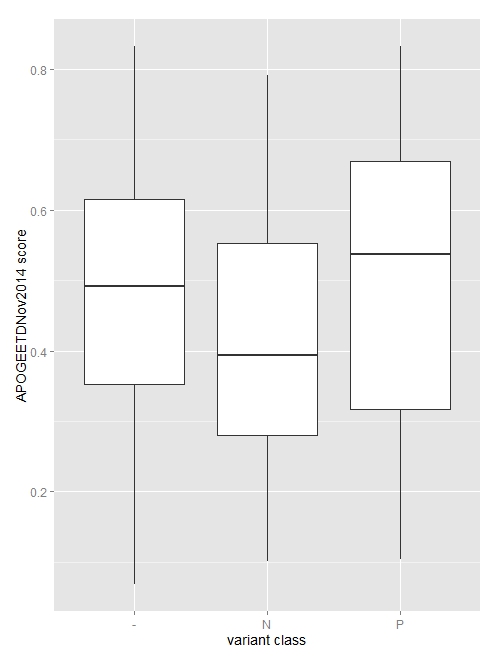


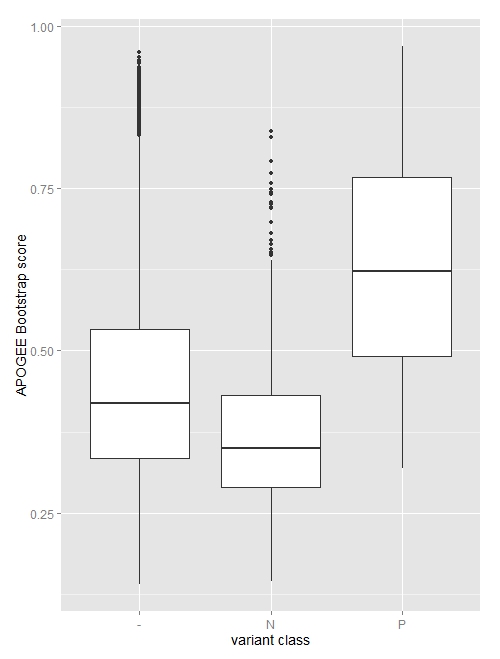

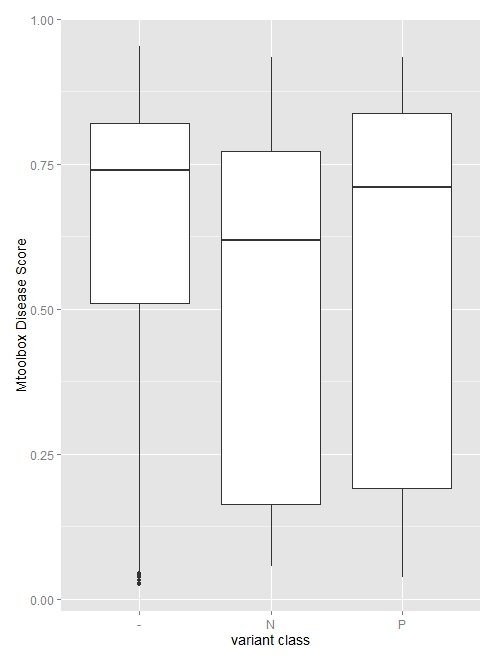


# Pairwise agreement assessment between categorical pathogenicity predictions

Pairwise comparisons of predictions revealed that there is good agreement between SIFT and FatHmm (86.81%), PROVEAN and MutationAssessor (81.94%), PROVEAN and PhD-SNP (81.37%), PROVEAN and SNAP (81.25%), MutationAssessor and PhD-SNP (82.06%), MutationAssessor and SNAP (84.39%), PhD-SNP and SNAP (80.9%). PANTHER was the most discordant (Table 3). The outcomes of the meta-predictors were generally poorly congruent. In particular, Condel was mostly in disagreement with all the others (Table 4).

| **Predictor** | PolyPhen2 | PolyPhen2b | SIFT | FatHmm | FatHmm_W | PROVEAN | MutationAssessor | EFIN (HD) | EFIN (SP) | CADD | PANTHER | PhD-SNP | SNAP |
| --- | --- | --- | --- | --- | --- | --- | --- | --- | --- | --- | --- | --- | --- |
| PolyPhen2 | - | - | 59,72 | 54,75 | 70,25 | 74,54 | 76,16 | 76,04 | 68,29 | 63,43 | 58,10 | 70,72 | 75,93 |
| PolyPhen2b |  | - | 52,08 | 43,17 | 65,86 | 75,46 | 78,13 | 68,40 | 64,00 | 63,19 | 56,94 | 74,19 | 76,39 |
| SIFT |  |  | - | 86,81 | 71,30 | 55,32 | 57,75 | 75,12 | 73,26 | 69,79 | 56,48 | 51,97 | 59,84 |
| FatHmm |  |  |  | - | 71,53 | 49,54 | 48,84 | 75,35 | 73,73 | 74,88 | 53,13 | 43,63 | 51,39 |
| FatHmm_W |  |  |  |  | - | 69,44 | 69,91 | 71,41 | 74,19 | 70,95 | 62,73 | 64,24 | 72,22 |
| PROVEAN |  |  |  |  |  | **-** | 81,94 | 63,77 | 68,63 | 60,53 | 59,61 | 81,37 | 81,25 |
| MutationAssessor |  |  |  |  |  |  | - | 66,09 | 71,99 | 59,84 | 60,07 | 82,06 | 84,38 |
| EFIN (HD) |  |  |  |  |  |  |  | - | 67,59 | 65,05 | 58,45 | 62,73 | 67,01 |
| EFIN (SP) |  |  |  |  |  |  |  |  | - | 68,52 | 58,68 | 58,33 | 72,11 |
| CADD |  |  |  |  |  |  |  |  |  | - | 50,58 | 55,32 | 61,00 |
| PANTHER |  |  |  |  |  |  |  |  |  |  | - | 50,58 | 61,92 |
| PhD-SNP |  |  |  |  |  |  |  |  |  |  |  | - | 80,90 |
| SNAP |  |  |  |  |  |  |  |  |  |  |  |  | - |

| **Meta-predictor** | CAROL | Condel | COVEC WMV | Meta-SNP | MToolBox DS | APOGEE Bootstrap |
| --- | --- | --- | --- | --- | --- | --- |
| CAROL | - | 15,51 | 86,81 | 75,69 | 81,25 | 60,03 |
| Condel |  | - | 13,54 | 23,38 | 8,33 | 41,2 |
| COVEC WMV |  |  | - | 80,32 | 81,36 | 65,28 |
| Meta-SNP |  |  |  | - | 76,38 | 60,64 |
| MToolBox DS |  |  |  |  | - | 55,09 |
| APOGEE Bootstrap |  |  |  |  |  | - |

# Details of APOGEE logistic model trees

The logistic model trees corresponding to the three versions of APOGEE (TDNov2014, TDJan2015 and TDJuly2015). They combine, in different manners, the PhyloP_100V and PhastCons_100V evolutionary conservation indexes, together with the PolyPhen2, SIFT, FatHmm_W, PROVEAN, MutationAssessor, EFIN_SP, EFIN_HD, CADD_phred, PANTHER, PhD-SNP and SNAP functional impact scores.

For each version, the number of logistic trees on the leaves, along with the logistic regression functions, are provided. The names of the selected predictors are reported within square brackets.

Training dataset: TDNov2014

Number of Leaves : 1

Size of the Tree : 1

LM_1:

Class 0 : 0.02 + [MutAss_score] * -0.17 + [EFIN_SP_score] * 0.67

Class 1 : -0.02 + [MutAss_score] * 0.17 + [EFIN_SP_score] * -0.67

Training dataset: TDJan2015

Number of Leaves : 3

Size of the Tree : 5

EFIN_SP_score <= 0.29: LM_1:3/6 (49)

EFIN_SP_score > 0.29

| MutAss_score <= 4.1: LM_2:3/9 (403)

| MutAss_score > 4.1: LM_3:3/9 (53)

LM_1:

Class 0 :

0.14 + [MutAss_score] * -0.1 + [EFIN_SP_score] * 1.08 + [CADD_phred_score] * -0.09 + [PANTHER_score] * 1.21 + [PhD-SNP_score] * -1.44

Class 1 :

-0.14 + [MutAss_score] * 0.1 + [EFIN_SP_score] * -1.08 + [CADD_phred_score] * 0.09 +[PANTHER_score] * -1.21 + [PhD-SNP_score] * 1.44

LM_2:

Class 0 :

0.06 + [PROVEAN_score] * 0.03 + [MutAss_score] * -0.1 +[EFIN_SP_score] * 0.36 +

[PANTHER.score] * 0.04 + [PhD-SNP_score] * 0.57

Class 1 :

-0.06 + [PROVEAN_score] * -0.03 + [MutAss_score] * 0.1 + [EFIN_SP_score] * -0.36 + [PANTHER.score] * -0.04 + [PhD-SNP_score] * -0.57

LM_3:

Class 0 :

4.88 + [PolyPhen2_score] * -2.35 + [SIFT_score] * -1.78 + [MutAss_score] * -0.1 + [EFIN_SP_score] * 0.36 + [PANTHER.score] * -0.33 + [PhD-SNP_score] * -2.97

Class 1 :

-4.88 + [PolyPhen2_score] * 2.35 + [SIFT_score] * 1.78 + [MutAss_score] * 0.1 + [EFIN_SP_score] * -0.36 + [PANTHER.score] * 0.33 + [PhD-SNP_score] * 2.97

Number of Leaves : 1

Training dataset: TDJul2015

Number of Leaves : 1

Size of the Tree : 1

LM_1:

Class 0 :

0.09 + [PhyloP_100V] * 0 + [PhastCons_100V] * -0.26 + [PolyPhen2_score] * 0.09 + [SIFT_score] * 0.14 + [FatHmmW_score] * -0.01 + [PROVEAN_score] * -0.02 + [MutAss_score] * -0.09 + [EFIN_SP_score] * 1.28 + [EFIN_HD_score] * -0.44 +[CADD_Phred_score] * -0.02 + [PANTHER.score] * -0.04 + [PhD-SNP_score] * 0.44 + [SNAP_score] * -0.44

Class 1 :

-0.09 + [PhyloP_100V] * 0 + [PhastCons_100V] * 0.26 + [PolyPhen2_score] * -0.09 + [SIFT_score] * -0.14 + [FatHmmW_score] * 0.01 + [PROVEAN_score] * 0.02 + [MutAss_score] * 0.09 + [EFIN_SP_score] * -1.28 + [EFIN_HD_score] * 0.44 + [CADD_phred_score] * 0.02 + [PANTHER_score] * 0.04 + [PhD-SNP_score] * -0.44 + [SNAP_score] * 0.44

The LMT models generated during all the 100 iterations can be downloaded [here](http://mitimpact.css-mendel.it/cdn/BoostrapStrategy_Models.zip).

# Comparison of datasets of known pathogenic variants

We retrieved all variants tagged as “disease” (n=58, 51 distinct amino acid changes) from Humsavar (06_2016 version) and verified that 48 were included in TDJul2015. These (overlapping) and those not present in TDJul2015 (non-overlapping) were mostly classified as deleterious by APOGEE (columns 4 and 5 in the table below). Contrary to non-overlapping variants, overlapping variants with Table 1 of ^2^ were mostly correctly predicted by APOGEE (31 out of 35, 88.6%). Furthermore, we compared the set of pathogenic variants of MToolBox with that of Humsavar: 20 deleterious non-synonymous variants were in common, 19 of which had an MToolBox DS < 0.4311 (thus classified as deleterious by that tool); 20/20 were deemed pathogenic by APOGEE.

| Resource | # of mapped  pathogenic variants | Presence in MitImpact TDJul2015 | APOGEE Bootstrap prediction for non-overlapping variants | APOGEE Bootstrap prediction for overlapping variants |
| --- | --- | --- | --- | --- |
| Humsavar_06_2016 | 58 | 48/58 | 10 P / 0 N (tot=10) | 46 P / 2 N (tot=48) |
| MToolBox Table1 | 53 | 35/53 | 10 P / 8 N (tot=18) | 31 P / 4 N (tot=35) |

List of MitImpact IDs of non-overlapping variants:

- Humsavar: *MI.20036, MI.20037, MI.20759, MI.24027, MI.2677, MI.4816, MI.7128, MI.8253, MI.8254, MI.946.*
- MToolBox: *MI.10601, MI.10661, MI.10939, MI.11528, MI.12052, MI.12511, MI.13528, MI.13823, MI.15906, MI.17828, MI.18114, MI.19045, MI.20233, MI.21041, MI.21834, MI.22474, MI.2545, MI.3290.*

List of MitImpact IDs of common variants:

- Humsavar: *MI.10598, MI.10948, MI.10950, MI.11080, MI.12086, MI.1224, MI.13701, MI.1384, MI.1401, MI.14380, MI.14851, MI.15172, MI.15248, MI.15259, MI.16101, MI.17005, MI.1789, MI.18291, MI.18470, MI.20030, MI.20173, MI.20343, MI.20753, MI.20760, MI.20840, MI.20860, MI.21768, MI.22186, MI.22231, MI.23726, MI.23738, MI.23786, MI.23787, MI.23790, MI.23799, MI.23813, MI.23816, MI.23971, MI.24026, MI.2779, MI.3124, MI.5365, MI.7905, MI.8247, MI.9409, MI.9930, MI.995, MI.997.*
- MToolBox: *MI.11080, MI.11443, MI.11568, MI.11574, MI.11638, MI.11964, MI.12032, MI.12093, MI.12561, MI.12664, MI.1384, MI.1385, MI.1401, MI.15172, MI.15248, MI.15259, MI.16101, MI.18467, MI.18470, MI.20030, MI.20753, MI.21239, MI.21768, MI.21770, MI.23738, MI.23786, MI.23787, MI.23790, MI.23799, MI.23813, MI.23971, MI.665, MI.9312, MI.995, MI.997.*

# Availability and query-ability of data

APOGEE was made freely accessible through an improved web interface to MitImpact, a collection of pre-computed pathogenicity predictions for all nucleotide changes that cause non-synonymous substitutions in human mitochondrial protein coding genes. It is implemented in Bootstrap, the most popular framework for developing responsive, mobile first projects on the web. The main inherited characteristic is the capability of efficiently scaling the web site from phones, to tablets, to desktops. Data can be downloaded freely as bulk, tab-separated, files. Alternatively, information on variants can be retrieved either by the GUI or, programmatically, by a new RESTful interface.

The GUI is designed to search a mutation by its genomic location, dbSNP identifier or relative protein position. Gene or protein names can be specified either as gene symbols or as Ensembl, Uniprot, NCBI identifiers. The RESTfull interface is implemented to react to HTTP requests occurring at:

mitimpact.css-mendel.it/api/v2.0/TYPE/PARAMS

where TYPE can be one of the following reserved words: genomic_position, protein_position or dbsnp. Parameters accepted by genomic_position are (comma-separated lists of) numbers or range of numbers (e.g., 3307-3309). dbsnp accepts individual or comma-separated lists of dbSNP ID (e.g, rs3020563, rs28520706, rs1041870). Setting TYPE to protein_position, instead, 2 parameters must be arranged in the following URL:

mitimpact.css-mendel.it/api/v2.0/protein_position?id=ID&pos=POS

where ID must be an actual comma-separated list of identifiers of the gene or of the protein of interest and POS must be a comma-separated list or range of integer numbers that specify the positions of the variants.

MitImpact can also be queried in the opposite manner, namely asking for all variants in a gene with a minimum number of pathogenic predictions. In particular,

mitimpact.css-mendel.it/api/v2.0/pathogenicity?id=ID&min=MIN,

where ID can be any mitochondrial gene or protein identifier and MIN specifies the minimum number of pathogenic assessments that a variant must have. This function queries: PolyPhen2, SIFT, FatHmm, FatHmm_W, PROVEAN, MutationAssessor, EFIN SP, EFIN HD, CADD, PANTHER, PhD-SNP and SNAP.

mitimpact.css-mendel.it/api/v2.0/consensus_pathogenicity?id=ID&min=MIN

This function queries the meta-predictors: Meta-SNP, CAROL, Condel, COVEC WMV, MToolBox and APOGEE. The output of any of these rest queries is formatted in JSON.

**References**

1. Castellana, S., Ronai, J. & Mazza, T. MitImpact: an exhaustive collection of pre-computed pathogenicity predictions of human mitochondrial non-synonymous variants. *Hum Mutat* **36**, E2413-22 (2015).

2. Santorsola, M. *et al.* A multi-parametric workflow for the prioritization of mitochondrial DNA variants of clinical interest. *Hum Genet* **135**, 121-36 (2016).
